# OsCESA9 conserved‐site mutation leads to largely enhanced plant lodging resistance and biomass enzymatic saccharification by reducing cellulose DP and crystallinity in rice

**DOI:** 10.1111/pbi.12700

**Published:** 2017-03-15

**Authors:** Fengcheng Li, Guosheng Xie, Jiangfeng Huang, Ran Zhang, Yu Li, Miaomiao Zhang, Yanting Wang, Ao Li, Xukai Li, Tao Xia, Chengcheng Qu, Fan Hu, Arthur J. Ragauskas, Liangcai Peng

**Affiliations:** ^1^ Biomass and Bioenergy Research Centre Huazhong Agricultural University Wuhan China; ^2^ National Key Laboratory of Crop Genetic Improvement Huazhong Agricultural University Wuhan China; ^3^ College of Plant Science and Technology Huazhong Agricultural University Wuhan China; ^4^ Key Laboratory of Crop Physiology, Ecology, Genetics and Breeding Ministry of Agriculture Rice Research Institute Shenyang Agricultural University Shenyang China; ^5^ College of Life Science and Technology Huazhong Agricultural University Wuhan China; ^6^ State Key Laboratory of Agricultural Microbiology Huazhong Agricultural University Wuhan China; ^7^ Department of Chemical and Biomolecular Engineering The University of Tennessee‐ Knoxville Knoxville TN USA; ^8^ Department of Forestry The University of Tennessee‐Knoxville Knoxville TN USA

**Keywords:** biomass saccharification, cellulose, CESA, lodging resistance, rice

## Abstract

Genetic modification of plant cell walls has been posed to reduce lignocellulose recalcitrance for enhancing biomass saccharification. Since cellulose synthase (CESA) gene was first identified, several dozen *CESA* mutants have been reported, but almost all mutants exhibit the defective phenotypes in plant growth and development. In this study, the rice (*Oryza sativa*) *Osfc16* mutant with substitutions (W481C, P482S) at P‐CR conserved site in CESA9 shows a slightly affected plant growth and higher biomass yield by 25%–41% compared with wild type (Nipponbare, a *japonica* variety). Chemical and ultrastructural analyses indicate that *Osfc16* has a significantly reduced cellulose crystallinity (CrI) and thinner secondary cell walls compared with wild type. CESA co‐IP detection, together with implementations of a proteasome inhibitor (MG132) and two distinct cellulose inhibitors (Calcofluor, CGA), shows that CESA9 mutation could affect integrity of CESA4/7/9 complexes, which may lead to rapid CESA proteasome degradation for low‐DP cellulose biosynthesis. These may reduce cellulose CrI, which improves plant lodging resistance, a major and integrated agronomic trait on plant growth and grain production, and enhances biomass enzymatic saccharification by up to 2.3‐fold and ethanol productivity by 34%–42%. This study has for the first time reported a direct modification for the low‐DP cellulose production that has broad applications in biomass industries.

## Introduction

Cellulose is the most abundant biomass convertible for biofuels and chemical products. As a principal component of plant cell walls, cellulose plays a central role in plant mechanical strength and morphogenesis (Somerville, 2006), but its features determine lignocellulose recalcitrance, leading to a costly biomass process (Himmel *et al*., 2007; Pauly and Keegstra, 2008). To reduce recalcitrance, genetic modifications of wall polymers (hemicelluloses and lignin) have been applied to enhance biomass saccharification (Bonawitz *et al*., 2014; Chen and Dixon, 2007; Chiniquy *et al*., 2012; Ding *et al*., 2012; Li *et al*., 2015; Wilkerson *et al*., 2014), but little has been reported about a direct alteration of cellulose in plants (Burton and Fincher, 2014).

Cellulose consists of β‐1,4‐linked glucan chains that form microfibrils by intra‐ and intermolecular hydrogen bonds. The formed hydrogen bonds significantly determine cellulose crystallinity, which is reportedly a key parameter negatively affecting biomass digestibility (Harris *et al*., 2012; Li *et al*., 2013; Zhang *et al*., 2013). The crystallinity index (CrI) has been broadly used to account for cellulose crystallinity and could be detected by X‐ray diffraction (XRD) patterns (Segal *et al*., 1959). Besides cellulose crystallinity, the degree of polymerization (DP) of crystalline cellulose is also regarded as an important cellulose feature (Zhang *et al*., 2013). Recent reports have indicated that cellulose CrI is positively correlated with its DP in *Miscanthus* samples (Zhang *et al*., 2013), and both cellulose features (CrI, DP) are the main factors that could negatively affect either plant lodging resistance or biomass enzymatic saccharification in plants (Li *et al*., 2015; Zhang *et al*., 2013). However, it remains largely unknown how cellulose biosynthesis process determines the cellulose features in plants.

In higher plants, cellulose is synthesized at the plasma membrane by cellulose synthase (*CESA*) enzymes that are organized into cellulose synthase complexes (CSCs) (Taylor *et al*., 2003). Since the first higher plant cellulose synthase gene was cloned from cotton in 1996 (Pear *et al*., 1996), the CESA superfamily has been characterized with eight transmembrane domains and a central cytoplasmic domain with D,D,D,QXXRW motif. The central cytoplasmic domain contains the plant‐conserved region (P‐CR) and class‐specific region (CSR), which may play a role in CESA protein association and assembly (Olek *et al*., 2014; Sethaphong *et al*., 2013). To dissect CESA biological functions, more than fifty distinct *CESA* mutants have been identified in different plant species through multiple genetic approaches (Table (Figure S1). Nevertheless, almost all mutants exhibit markedly reduced cellulose and defective growth phenotypes, and several mutants are examined with low cellulose crystallinity for high biomass enzymatic digestibility (Table (Figure S1). To our knowledge, however, little is yet reported about cellulose DP alteration from the mutants. Furthermore, the homologous and heterologous overexpression of *CESA* genes could not enhance cellulose products but did affect plant growth in transgenic plants (Table (Figure S1). Exceptionally, the recent rice *bc13* mutant with one amino acid alteration in CESA9 showed normal plant growth and cadmium tolerance, despite a reduction in cellulose (Song *et al*., 2013).

Rice is a major food crop over the world with enormous biomass residues for biofuels and chemical products. In this study, we identified a novel rice CESA9 allele *Osfc16* that showed a normal plant growth and high biomass production. Mutation of the CESA9 protein reduced two cellulose features (CrI, DP), leading to improved plant lodging resistance and enhanced biomass enzymatic saccharification. Further analysis revealed that the P‐CR region mutation of CESA9 protein could affect stability of secondary wall CSCs, which may early terminate the CSC track in the plasma membrane resulting in low‐DP cellulose synthesis.

## Results

### CESA9 conserved‐site mutation and improved agronomic traits in *Osfc16*

**Figure 1:**
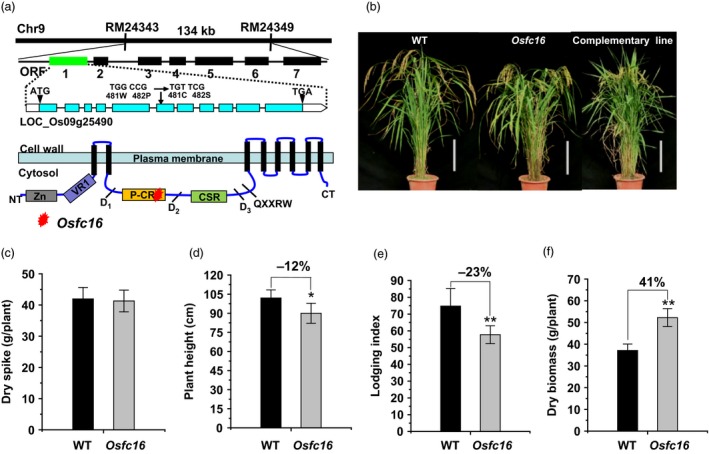
*Osfc16* mutant identification and agronomic trait observation. (a) Location of *Osfc16* mutation with substitutions of Trp and Pro residues with Cys and Ser at the 481 and 482 position of the CESA9 protein. (b) Plant growth in wild‐type (WT), *Osfc16* mutant and complementary line (scale bar = 20 cm). (c) Dry spike. (d) Plant height. (e) Lodging index. (f) Dry biomass. * and ** indicate significant differences between WT and *Osfc16* mutant by *t*‐test at *P *<* *0.05 and 0.01, respectively, with the increased or decreased percentage (%) calculated by subtraction of the values between mutant and WT divided by WT. The error bar indicates SD values (*n* = 3).

Using map‐based cloning approach, the rice *Osfc16* mutant was identified as a single recessive gene, which encodes the CESA9 protein with two amino acid substitutions (**W** ^481^ **P** ^482^GN→**C** ^481^ **S** ^482^GN) in the site of P‐CR region (Figure 1a). In particular, the substituted amino acids (Trp and Pro) are fully conserved in all CESA family proteins of the eight plant species examined (Figure S1). Although several dozens of *CESA* mutants and overexpressed transgenic plants have been previously identified with remarkably defective phenotypes in different plant species (Table (Figure S1), the *Osfc16* mutant exhibited a normal plant growth as observed in wild type (Nipponbare (NPB), a *japonica* variety) (Figure 1b). In 3‐year (2012–2014) independent field experiments, the *Osfc16* mutant maintained grain yields (dry spike) similar to wild type (Figure 1c and Table (Figure S2). Notably, despite the relatively short height (Figure 1d), the *Osfc16* mutant had significantly improved plant lodging resistance (lodging index reduced by 18%–24%) and enhanced biomass production (dry straw increased by 25%–41%), compared with wild type (Figure 1e,f and Table S2). In particular, tillers numbers (tillers/plant) were much increased in the *Osfc16* mutant by 59%–68%, attributing for its higher biomass production (Table S2).

To verify the *Osfc16* mutation as the single recessive gene, the full‐length cDNA of *CESA9* gene was expressed in the *Osfc16* mutant. As a result, the *Osfc16* mutant phenotype was fully complemented (Figure 1b), and the related major agronomic traits (lodging index and dry straw) were restored in three independent complementary transgenic lines at significant levels (Table S3).

### Enhanced biomass saccharification and ethanol production in *Osfc16*

**Figure 2:**
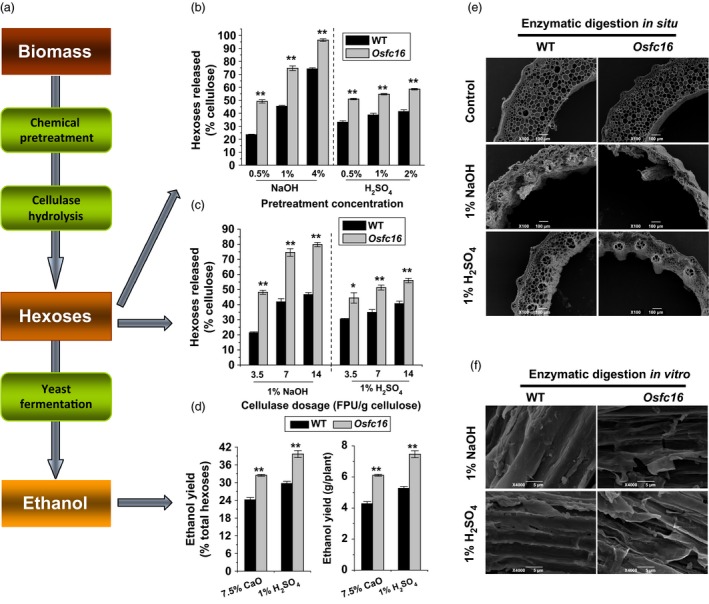
Biomass enzymatic saccharification and ethanol production. (a) Scheme for biomass enzymatic saccharification and ethanol yield. (b) Hexose yields released from enzymatic (mixed‐cellulase) hydrolysis after pretreatment with NaOH and H_2_ SO_4_ at three concentrations. (c) Hexose yields released from three dosages of mixed‐cellulase hydrolysis after pretreatment with 1% NaOH and 1% H_2_ SO_4_. (d) Ethanol yield obtained by yeast fermentation of the sugars from biomass enzymatic hydrolysis of the mature stems after pretreatment with 7.5% CaO or 1% H_2_ SO_4_. Ethanol yield was expressed as either percentage of total hexoses in the biomass residues or ethanol yield per plant. (e) SEM images of *in situ* enzymatic digestion of stems at heading stage after 1% NaOH or 1% H_2_ SO_4_ pretreatment and sequential enzymatic hydrolysis. (f) SEM images of *in vitro* enzymatic digestion of biomass residues released from enzymatic hydrolysis after 1% NaOH or 1% H_2_ SO_4_ pretreatment. * and ** indicate significant differences between WT and *Osfc16* mutant by *t*‐test at *P *<* *0.05 and 0.01, respectively, and the error bar indicates SD values (*n* = 3).

Using mature stem materials, we detected biomass enzymatic digestibility (saccharification) in the *Osfc16* mutant by calculating the hexose yields released from enzymatic hydrolysis of pretreated biomass (Figure 2a). The *Osfc16* mutant exhibited higher yields of hexoses by up to 2.3‐fold than that of wild type, under pretreatments with three concentrations of alkali (0.5%, 1% and 4% NaOH) and acid (0.5%, 1% and 2% H_2_SO_4_) or upon enzymatic hydrolysis with three dosages of cellulase (3.5, 7 and 14 FPU/g cellulose) (Figure 2b,c; Figure (Figure S2; Table S4). Such large enhancements were confirmed by visualizations of more violent destruction of stem tissue *in situ* (Figure 2e) and of rougher biomass residue surfaces *in vitro* (Figure 2f) in the *Osfc16* mutant from 1% NaOH and 1% H_2_SO_4_ pretreatments and sequential enzymatic hydrolyses. Furthermore, the *Osfc16* mutant, compared with wild type, exhibited higher ethanol yields by 34%–42% obtained by yeast fermentation of the sugars released from biomass enzymatic hydrolysis of rice straw upon the mild chemical (7.5% CaO, 1% H_2_SO_4_) pretreatments (Figure 2d; Table S5). This study demonstrated that the CESA9 site mutation could lead to largely enhanced biomass saccharification and ethanol productivity in the *Osfc16* mutant.

### Altered cell wall composition and structure in *Osfc16*

**Figure 3:**
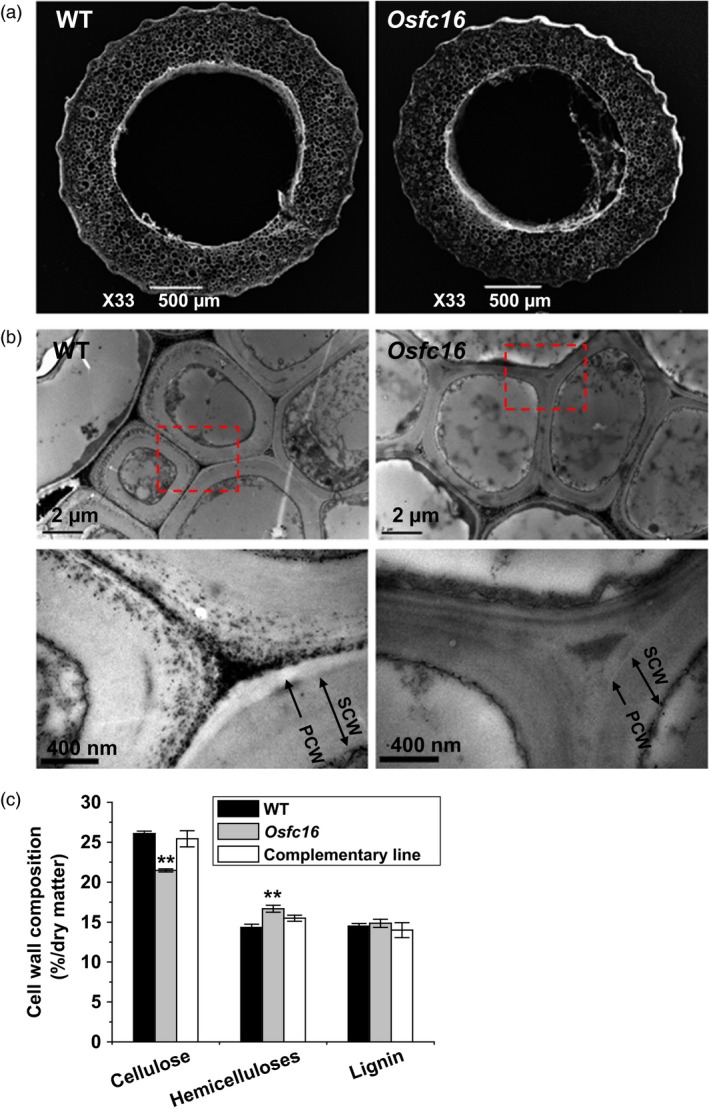
Observations of stem tissues and cell wall structures. (a) SEM images of the second‐internode stem at the heading stage of rice. (b) TEM images of the sclerenchyma cell walls. PCW: primary cell wall. SCW: secondary cell wall. (c) Cell wall composition of mature stems. ** indicates significant differences between WT and *Osfc16* or complementary line by *t*‐test at *P *<* *0.01, and the error bar indicates SD values (*n* = 3).

To understand the improved agronomic traits and enhanced biomass digestibility in *Osfc16* mutant, we examined its cell wall composition and structure. Besides relatively smaller‐diameter stems (Figure 3a), the *Osfc16* mutant showed thinner secondary cell walls than wild type (Figure 3b). Chemical analysis indicated that the *Osfc16* mutant had reduced cellulose levels by 18% and increased hemicellulose levels by 16% with lignin level similar to wild type in the mature stems (Figure 3c). Furthermore, the *Osfc16* mutant did not show much difference from wild type in monosaccharide composition of hemicelluloses and three monomer constituents (G, S and H) of lignin (data not shown). In addition, the cell wall composition of *Osfc16* mutant could be fully restored in three independent complementary transgenic lines.

### Reduced cellulose crystallinity in *Osfc16*

**Figure 4:**
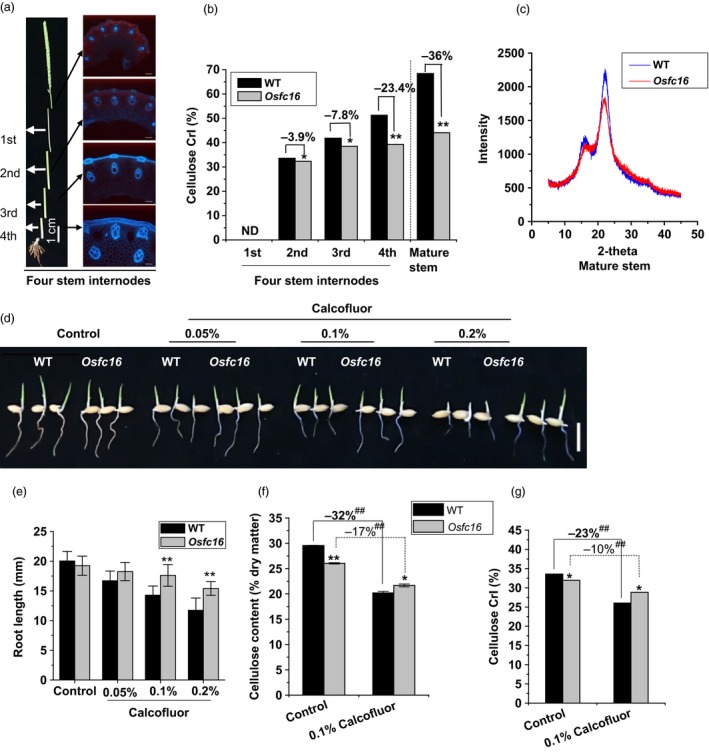
Detection of cellulose crystallinity. (a) Four‐internode stems at heading stage used for CrI and DP detection. (b) Cellulose CrI of the four internodes and mature stems using the X‐ray diffraction (XRD) method. (c) The XRD scanning patters applied for CrI calculation. (d, e) Root lengths of the germinated seedlings treated with Calcofluor for 48 h. (f, g) Cellulose content and CrI in roots of the seedlings treated with 0.1% Calcofluor for 48 h. * and ** indicate significant differences between WT and *Osfc16* by *t*‐test at *P *<* *0.05 and 0.01, respectively, with the increased or decreased percentage (%) calculated by subtraction of the values between mutant and WT divided by WT. The error bar indicates SD values. ^##^ indicates significant differences between the Calcofluor treatment and control by *t*‐test at *P *<* *0.01, with the increased or decreased percentage (%) calculated by subtraction of the values between the Calcofluor and control divided by control.

As a major cellulose feature, cellulose crystallinity has been characterized by determining crystalline index (CrI) of biomass samples (Li *et al*., 2015; Xu *et al*., 2012; Zhang *et al*., 2013). Using four internodes of stems at heading stage of rice (Figure 4a), a standard development from primary to secondary cell walls (Xie *et al*., 2013), the *Osfc16* mutant exhibited a significant reduction of the cellulose CrI in the second, third and fourth internodes by 3.9%, 7.8% and 23.4%, respectively, compared with wild type (Figure 4b). Notably, the *Osfc16* mutant had much lower CrI value than wild type by 36% in the mature stem that is rich in secondary cell walls (Figure 4b,c). Because slight different CrI values were detected between *Osfc16* and wild type in the second‐internode stems that are predominately composed of primary cell walls, the data thus indicated that a major reduction of cellulose CrI occurred in the secondary cell walls of *Osfc16* mutant, consistent with its thinner secondary cell walls.

**Figure 5:**
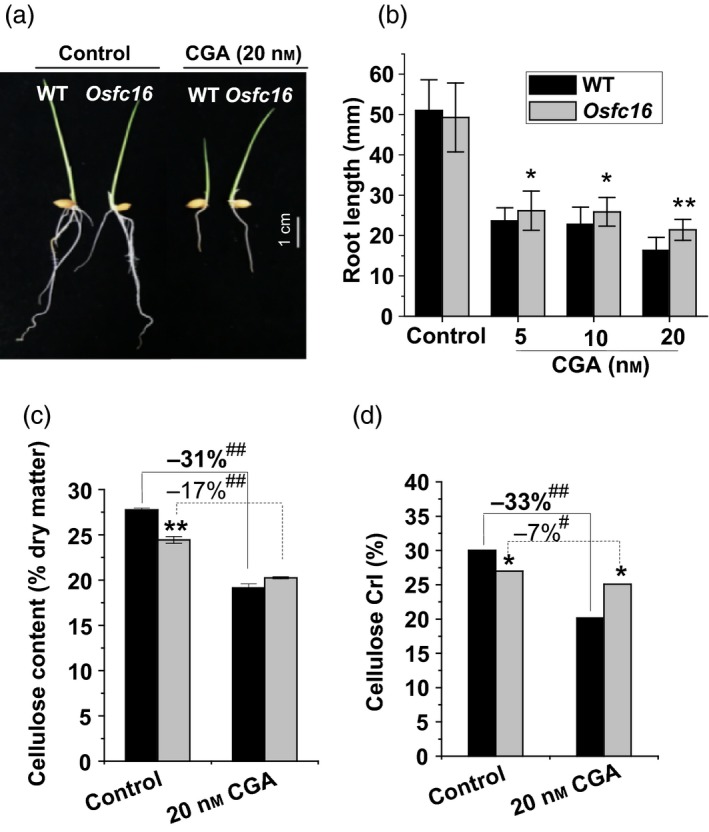
CGA effects on plant growth and cellulose crystallinity. (a, b) Root lengths of the germinated seeds treated with CGA for 72 h. (c, d) Cellulose content and CrI in roots of the seedlings treated with 20 nM CGA for 72 h. * and ** indicate significant differences between WT and *Osfc16* mutant by *t*‐test at *P *<* *0.05 and 0.01, respectively, and the error bar indicates SD (*n* = 3). ^#^ and ^##^ indicate significant differences between the CGA treatment and control by *t*‐test at *P *<* *0.05 and 0.01, respectively, with the increased or decreased percentage (%) calculated by subtraction of the values between CGA and control divided by control.

To further confirm the reduction of cellulose CrI in *Osfc16* mutant, we applied two distinct cellulose inhibitors (Calcofluor, CGA325′615‐CGA) to treat with rice seedlings. While the germinated rice seeds were incubated with Calcofluor, an inhibitor of cellulose crystallization (Haigler *et al*., 1980), the *Osfc16* mutant showed less retarded root growth than did the wild type (Figure 4d,e and Table S6). As Calcofluor influences microfibril crystallization by competing for hydrogen binding sites that form the crystalline lattice (Haigler *et al*., 1980), the *Osfc16* mutant, which is rich in low‐CrI cellulose, should have less binding capability with Calcofluor, ultimately leading to less inhibited plant growth and relatively higher cellulose level and CrI value, compared with wild type (Figure 4f,g). Furthermore, while treated with CGA, the *Osfc16* mutant also showed much less retarded root growth and reduced cellulose level, compared with wild type (Figure 5a–c and Table S6). Notably, the *Osfc16* mutant treated with CGA had a significantly higher CrI value than wild type (Figure 5d), a similar phenomenon observed in the Calcofluor treatment (Figure 4g). Because CGA is presumed to affect CESA complex association on the plasma membrane (Crowell *et al*., 2009; Kurek *et al*., 2002; Peng *et al*., 2001, 2002), the wild type may be much more affected by CGA to produce low‐CrI cellulose (Figure 5d), whereas the *Osfc16* mutant was less sensitive to CGA, probably due to its unstable CESA complexes as described below. Hence, in terms of its low sensitivity to two distinct cellulose inhibitors, the *Osfc16* mutant had much less reduction of cellulose CrI by 10% and 7% relative to the control, whereas the wild type showed the reduced CrI by 23% and 33% (Table S6), which on the contrary confirmed that the *Osfc16* mutant had a significantly reduced cellulose crystallinity.

### Reduced cellulose DP in *Osfc16*

**Figure 6:**
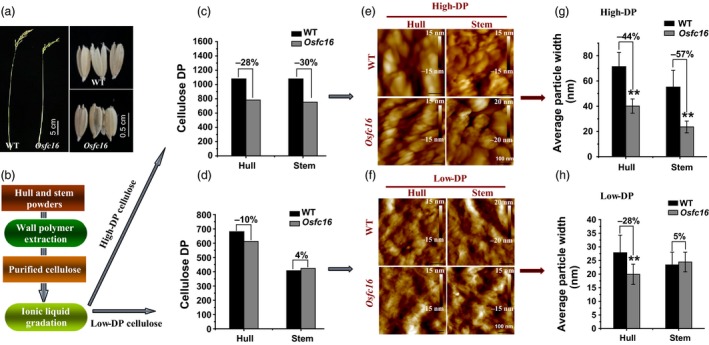
Measurements of cellulose DP in the gradated cellulose fractions of stem and hull tissues in wild type and *Osfc16* mutant. (a, b) Mature stem and hull tissues collected for cellulose extraction and gradation into high‐ and low‐DP cellulose fractions. (c, d) Detection of cellulose DP in the high‐ and low‐DP fractions from one independent biological experiment (Table S8). (e, f) AFM observation of cellulose surfaces in the high‐ and low‐DP fractions. (g, h) Quantitative analysis of AFM imagine by randomly selecting ten dots in the high‐ and low‐DP factions. ** indicates significant differences between the WT and *Osfc16* mutant by *t*‐test at *P *<* *0.01, with the increased or decreased percentage (%) calculated by subtraction of the DP values between WT and mutant divided by WT.

As cellulose CrI is positively correlated with its DP (Zhang *et al*., 2013), it remains essential to examine cellulose DP in the *Osfc16* mutant. In this study, we focused on detecting cellulose DP of stem and hull tissues in both *Osfc16* mutant and wild type (Figure 6a), because both tissues are of predominately secondary cell walls containing extremely high cellulose and lignin for biomass application (Table S7). However, to distinguish cellulose DP in primary and secondary cell walls, we established a novel approach to extract intact cellulose samples by fully removing hemicelluloses and lignin with 4 M KOH and 8% NaClO_2_ under mild conditions and consequently gradated the purified cellulose into relatively low‐ and high‐DP cellulose fractions using ionic liquid (1‐butyl‐3‐methylimidazolium acetate) and DMSO chemicals (Figure 6b). Using the viscometry method, a classic assay for cellulose DP (Kumar *et al*., 2009; Li *et al*., 2014; Zhang *et al*., 2013), we examined that the *Osfc16* mutant in the high‐DP cellulose fractions exhibited much lower cellulose DP values by 28%–30% than did the wild type in hull and stem tissues from two independent biological replicate experiments (Figure 6c and Table S8). By contrast, much different DP values were not determined between wild type and mutant in the low‐DP fractions (Figure 6d). These findings were confirmed by atomic force microscopy (AFM) observations in which the *Osfc16* mutant exhibited much smaller cellulose particles by 44%–57% than did wild types in the high‐DP fraction (Figure 6e,g). Because the high‐DP fractions cover 10%–40% of total cellulose in the hull and stem tissues (Table S8), their cellulose is thus derived from the secondary cell walls, whereas the low‐DP fractions should contain the cellulose from primary cell walls and partial secondary cell walls. Hence, the results indicated that the *Osfc16* mutant could partially synthesize the low‐DP cellulose in the secondary cell walls of hull and stem tissues, compared with wild type. In addition, although relatively small particles were observed in the low‐DP fractions, the *Osfc16* mutant had significantly smaller particles by 28% than did the wild type in the hull and in the stem (Figure 6f,h). It should explain that the hull contained much more cellulose from secondary cell walls than did the stem as described above (Table S7). Taken all together, the results demonstrated that the *Osfc16* mutant could synthesize low‐DP cellulose in the secondary cell walls, which should lead to thinner secondary cell wall and reduced cellulose level and CrI relative to wild type.

### Affected CESA4/7/9 complex association in *Osfc16*

**Figure 7:**
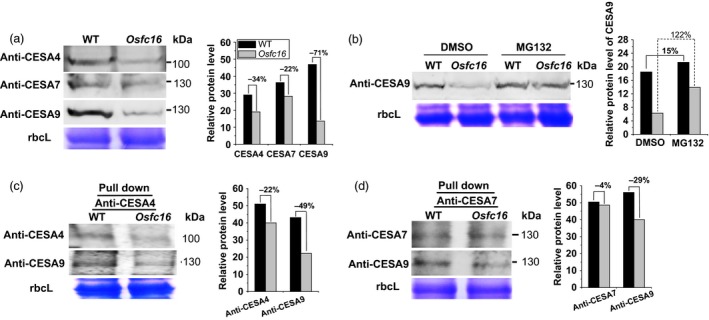
Western blot analysis of CESA proteins. (a) Detection of total CESA4, CESA7 and CESA9 proteins using microsomal membrane extracts of stems at heading stage. (b) CESA9 proteins in the stems treated with 150 μm MG132 and an equivalent dilution of DMSO (control) for 4 h. (c, d) CESA4, CESA7 and CESA9 proteins using co‐immunoprecipitation with anti‐CESA4 and anti‐CESA7. The decreased percentage (%) was calculated by subtraction of the relative protein levels between mutant and WT divided by WT.

Because the CESA4/7/9 are required to form a functional cellulose synthase complexes for secondary cell wall synthesis in rice (Huang *et al*., 2015; Liu *et al*., 2013; Tanaka *et al*., 2003; Wang *et al*., 2010), the three CESA proteins were detected by Western blot analysis of microsomal membrane extracts. Compared with wild type, the *Osfc16* mutant showed much lower CESA9 protein levels by 71% as well as reduced CESA4 and CESA7 protein levels by 34% and 22%, respectively (Figure 7a). To sort out the CESA9 protein reduction in the mutant, we used MG132, a proteasome inhibitor (Smalle and Vierstra, 2004), to treat rice plants at tillering stage (Figure 7b). When treated with MG132, both *Osfc16* and wild‐type plants exhibited higher CESA9 protein levels than did those treated only with DMSO (control), indicating that CESA9 is degraded in a proteasome‐dependent manner in plant cells. Notably, the *Osfc16* mutant treated with MG132 had increased CESA9 protein biosynthesis rates by onefold compared with the control, whereas wild type only showed biosynthesis rate that increased by 15%, suggesting a rapid and massive proteasome degradation of the CESA9 protein in the *Osfc16* mutant. Furthermore, we detected the levels of CESA9 in the CESA4/7/9 complexes pulled down by anti‐CESA4 and anti‐CESA7, respectively (Figure 7c,d). Although the levels of CESA4 and 7 proteins were reduced by 22% and 4%, the *Osfc16* mutant showed much lower CESA9 protein levels by 49% and 29% than did the wild type, indicating that the *Osfc16* mutant had reduced CESA9 in proportion to the CESA4/7/9 complexes. Therefore, the CESA9 conserved‐site mutation affects its association with the CESA complexes, leading to a rapid proteasome degradation. On the other hand, because CGA could affect CESA complex association, this result may also explain why the *Osfc16* mutant was less sensitive to CGA treatment than was the wild type as described above.

## Discussion

It has been defined that genetic modification of plant cell walls should not only enhance biomass enzymatic saccharification, but also have little effect on plant growth and development (Abramson *et al*., 2010). Although previous *CESA* mutation alleles exhibited enhanced biomass digestibility by reducing cellulose crystallinity, various defective plant growth phenotypes had been observed in almost all *CESA* mutants and *CESA*‐overexpressed transgenic plants (Table (Figure S1). Therefore, this study indicates a new genetic strategy on a direct cellulose modification by CESA mutation at plant fully conserved sites. As recent CRISPR/Cas9 technology is well developed (Doudna and Charpentier, 2014), it could be applied to generate a bunch of mutants from other conserved‐site mutations in three CESA4/7/9 isoforms, which may lead to finding out optimal mutants in rice and beyond. In addition, characterization of those generated mutants should further interpret why the *CESA* mutants with CESA9 conserved‐site mutations could maintain a normal plant growth and grain production in plants.

Notably, the *Osfc16* mutant has exhibited much higher biomass production and plant lodging resistance than did the wild type. As the plant height is negatively correlated with tiller number in rice (Li *et al*., 2003; Zhao *et al*., 2014), the relatively thin stems and short height of *Osfc16* mutant may cause its increased tiller number per plant for high biomass production. Plant lodging resistance is a major and integrated agronomic trait on plant growth and grain production (Li *et al*., 2015). In particular, rice lodging resistance is negatively affected with plant height and fresh weight (Crook and Ennos, 1994; Islam *et al*., 2007). Importantly, cellulose crystallinity has been recently demonstrated as the main factor negatively determining plant lodging resistance in rice (Li *et al*., 2015). Therefore, the *Osfc16* mutant showing much higher lodging resistance should be due to reductions of related factors, such as shorter height, less fresh weight per tiller and lower cellulose CrI. In addition, it remains interesting to test whether the CESA conserved‐site mutation could enhance lodging resistance in other plants.

Cellulose CrI reflects the relative amount of crystalline material in cellulose, and highly crystalline cellulose is less accessible to cellulase attack than amorphous cellulose on biomass hydrolysis (Himmel *et al*., 2007). However, cellulose DP is another important factor on biomass digestibility, because decreasing cellulose DP could increase both number of β‐1,4‐glucan chain‐reducing ends and proportion of amorphous cellulose. In this study, it has been demonstrated that the OsCESA9 site mutation could much reduce cellulose DP and CrI for largely enhanced biomass enzymatic saccharification in the *Osfc16* mutant, which is distinct from the lignin and hemicellulose modifications that increase biomass digestion by improving enzyme accessibility to the cellulose surface (Bonawitz *et al*., 2014; Chen and Dixon, 2007; Chiniquy *et al*., 2012; Ding *et al*., 2012; Li *et al*., 2015; Wilkerson *et al*., 2014). In addition, because hemicelluloses negatively affect cellulose crystallinity (Li *et al*., 2013; Xu *et al*., 2012), the relatively high level of hemicelluloses in the *Osfc16* mutant (Figure 3c) should be an additional contributor to its biomass enzymatic saccharification.

**Figure 8:**
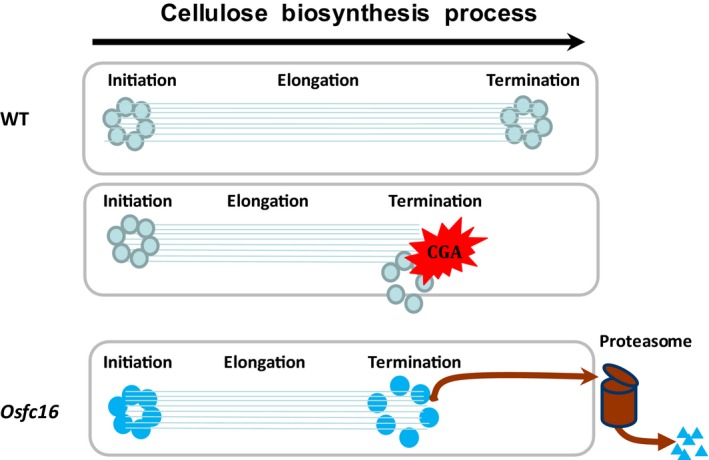
A hypothesis model on cellulose biosynthesis process involved in initiation, elongation and termination of β‐1,4‐glucan chains synthesized by CESA complexes on plasma membrane, which highlights that the CESA complexes in the *Osfc16* mutant or in the WT treated with CGA may have a reduced lifetime, leading to relatively early termination of β‐1,4‐glucan chains for low‐DP cellulose biosynthesis.

Plant cellulose biosynthesis process principally involves in three major steps: β‐1,4‐glucan chain initiation, elongation and termination (Peng *et al*., 2002). Although CESA complexes are presumed to synthesize the β‐1,4‐glucan chains, little is yet known about the chain termination that determines cellulose DP. Hence, this study proposed a hypothetic model that the low‐DP cellulose synthesis in *Osfc16* mutant should be due to the CESA9 site mutation that may reduce lifetime of CESA4/7/9 complexes towards a relatively early β‐1,4‐glucan chain termination (Figure 8). Here are four evidences: (i) CESA9 site mutation occurs in the P‐CR region that has been proposed to function in CESA protein association and assembly (Olek *et al*., 2014; Sethaphong *et al*., 2013); (ii) all three CESA4/7/9 proteins are reduced in the *Osfc16* mutant from co‐immunoprecipitation assays; (iii) *Osfc16* mutation mimics the CGA inhibition mode that could disassociate CESA complexes in plants; and (iv) *Osfc16* mutation leads to a rapid proteasome degradation of CESA proteins. On the other hand, as the Cys and Ser substitution with Trp and Pro in the *Osfc16* mutant may play a role in protein interaction and modification, it remains interesting to test whether both amino acids could affect CESA complex association by generating new mutants in the future.

In conclusion, the CESA9 conserved‐site mutation could affect its association with the CESA complexes towards a rapid proteasome degradation and cause the low‐DP cellulose synthesis for a reduced lignocellulose crystallinity, which largely enhances plant lodging resistance and biomass enzymatic saccharification in *Osfc16* mutant. In addition, this study provides the perspective to find out the optimal mutants from other conserved‐site mutations in CESA4/7/9 using CRISPR/Cas9 technique. It also suggests a potential genetic manipulation on the genes that could lead to defective phenotypes from overexpression and knockout in plants.

## Experimental procedures

### Plant sample collections and physical character measurements

The homozygous *Osfc16* mutant and wild‐type plants (*japonica* cultivar Nipponbare (NPB)) were respectively grown in the experimental fields of Huazhong Agricultural University, Wuhan, China, in 2012, 2013 and 2014. The collected mature stem tissues were dried at 55 °C, cut into small pieces, ground through 40‐mesh screen (0.425 × 0.425 mm) and stored in the dry container until use.

Rice dry spike and dry biomass were respectively weighed after the samples were dried in the oven at 60 °C. Plant lodging index was detected at six independent biological duplicates using the stem tissues at 30 days after flowering. The breaking resistance of the third internode was detected using a prostrate tester (DIK 7401, Japan), with the distance between fulcra of the tester at 5 cm. Fresh weight (W) of the upper portion of the plant was measured including panicle and the three internodes, leaf and leaf sheath. Bending moment (BM) and lodging index (LI) were calculated using the following formulae: BM = Length from the third internode to the top of panicle × W; and LI = BM/breaking resistance.

### Genetic identification of *Osfc16* mutant

The *Osfc16* mutant was selected in 2008 from *japonica* variety Nipponbare T‐DNA mutagenesis pools. To identify the *Osfc16* mutant, a F_2_ mapping population was generated from the crossing between *Osfc16* mutant and SH838, an *indica* fertility‐restoring line in China. The segregation ratio in F_2_ population showed that the normal plants and brittle culm plants segregated as 3 : 1. Map‐based cloning approach was then used for gene identification of the *Osfc16,* based on ~5000 F_2_ mutant plants with SSR molecular markers. The *Osfc16* gene was localized between RM24343 (forward primer: 5′‐AACTGCCACTGCCAATCATCG‐3′; and reverse primer: 5′‐CTCCAGCTCTCTCCACGACTCC‐3′) and RM24349 (forward primer: 5′‐GT ACTACTAGCTCGGCTGCTCTGC‐3′; and reverse primer: 5′‐GTAGTGGAGAGC GTGGACAGC‐3′) on chromosome 9 within a 134 kb in the rice genome (Figure 1a). The 134‐kb genomic region containing the *CESA9* gene was amplified from the mutants and their corresponding wild‐type plants by PCR with KOD‐Plus (TOYOBO, Japan) and sequenced with a 3730 sequencer (ABI, Massachusetts, USA).

For genetic complementation of *Osfc16* mutant, a 3401‐bp cDNA fragment containing the entire *CESA9* coding region driven by the *ubiquitin* promoter was cloned into the binary vector pCAMBIA 3300 to generate the binary plasmid with forward primer 5′‐CTTCTAGACTCCTCTCCTCCTTCCTGCGTC‐3′ and reverse primer 5′‐TTCCTGCAGGGCCATCTGTCCATTCCCTCTTC‐3′. This binary plasmid was introduced into *Agrobacterium tumefaciens* strain *EHA105* and transformed into the *Osfc16* mutant. The complementary transgenic plant lines of *Osfc16* mutant that expressed full‐length cDNA of wild‐type *CESA9* gene were characterized as shown in Figures 1b and 3c and Table S3. To examine the T‐DNA insertion in the *Osfc16* mutant, TAIL‐PCR was performed to analyse the genetic cosegregation of the mutant phenotype with T‐DNA insertion based on the known T‐DNA sequences. As a result, the T‐DNA insertion occurred in the nonfunctional gene and did not exhibit association with the brittle culm phenotypes.

### Plant cell wall fractionations

Plant cell wall fractionations were performed as described previously (Peng *et al*., 2000), with minor modifications as follows: the dry biomass powder (40 mesh) samples (0.1–1.0 g) were washed twice with 5.0 mL buffer and twice with 5.0 mL distilled water. The remaining pellet was stirred with 5.0 mL chloroform–methanol (1 : 1, v/v) for 1 h at 40 °C and washed twice with 5.0 mL methanol, followed by 5.0 mL acetone. The pellet was washed once with 5.0 mL distilled water. The remaining pellet was added with 5.0‐mL aliquot of DMSO–water (9 : 1, v/v), rocked gently on a shaker overnight. After centrifugation, the pellet was washed twice with 5.0 mL DMSO–water and then with 5.0 mL distilled water three times. The remaining pellet was defined as crude cell wall. The remaining crude cell wall was suspended in 0.5% (w/v) ammonium oxalate (5.0 mL) and heated for 1 h in a boiling water bath. During this step, the sample was stirred vigorously every 10 min to prevent the accumulation of materials at the tube surface. After centrifugation and washing the pellet once with 5.0 mL ammonium oxalate and twice with 5.0 mL distilled water, the pellet was suspended in 4 M KOH containing 1.0 mg/mL sodium borohydride (5.0 mL) and incubated for 1 h at 25 °C. During this step, the sample was stirred vigorously every 10 min. After centrifugation, the pellet was washed once with 5.0 mL 4 m KOH and twice with 5.0 mL distilled water. The remaining pellet was defined as crude cellulose. Meanwhile, the remaining pellet from KOH extraction (crude cellulose) was also suspended in 5.0 mL acetic acid–nitric acid–water (8 : 1 : 2, v/v/v) and heated for 1 h in a boiling water bath with stirring every 10 min. After centrifugation, the pellet was washed twice with 5.0 mL water and the remaining pellet was defined as crystalline cellulose sample.

### Cellulose extraction and gradation

The dry biomass powders (0.2–1 g) of hull and stem samples were treated with 4 m KOH containing 1.0 mg/mL sodium borohydride (10 mL) at 25 °C for 1 h and then centrifuged (2810 ***g***) for 5 min. The pellet was retreated with 4 m KOH for one more time and washed with distilled water five times until pH at 7.0. The remaining pellet was further added with 8% NaClO_2_ (10 mL) at 25 °C for 72 h (NaClO_2_ change every 12 h). After centrifugation, the pellet samples were washed with distilled water for five times until pH 7.0 and then further treated with 50 U xylanase (Lot 91101c; Megazyme, Ireland) at 60 °C for 24 h. The remaining pellet was retreated with 8% NaClO_2_ for one more time and dried as purified cellulose sample, which were verified with nondetectable lignin and less than 1%–2% (of dry matter) pentoses.

The purified cellulose samples (40 mg) were further treated with 3 mL 1‐butyl‐3‐methylimidazolium acetate at 70 °C for 25 min (stem) and 40 min (hull), respectively. The samples were added with 3 mL DMSO and then fully suspended. After centrifugation (2810 ***g***) for 5 min, the supernatant was collected as low‐DP cellulose sample for cellulose level assay and atomic force microscopy (AFM) observation as described below. The remaining pellet was retreated with 2 mL 1‐butyl‐3‐methylimidazolium acetate at 90 °C until fully dissolved, and then well mixed with 2 mL DMSO as collection of high‐DP cellulose sample for cellulose level assay and AFM observation. The high‐ and low‐DP cellulose samples were respectively mixed with distilled water (1 : 1, v/v) at 50 °C and centrifuged (2810 ***g***) for 5 min. The precipitated residues were then collected as high‐ and low‐DP cellulose samples for DP detection by the viscometry method described below.

### Cell wall composition determinations

Cellulose level was determined using the anthrone/H_2_SO_4_ method (Fry, 1988), and total hemicellulose contents were calculated subjective to total hexoses and pentoses in the hemicellulose fraction. Total pentoses were detected using the orcinol/HCl method (Dische, 1962). To eliminate the interference of pentoses on hexoses reading at 620 nm, a deduction from pentoses reading at 660 nm was carried out for final hexoses calculation. A standard curve referred for the deduction was drawn using a series of xylose concentrations, which was confirmed by GC‐MS analysis. Total lignin content was determined by the two‐step acid hydrolysis method according to the Laboratory Analytical Procedure of the National Renewable Energy Laboratory, USA (Sluiter *et al*., 2008). All experiments were conducted in the biological triplicates.

### Cellulose CrI and DP detections

The X‐ray diffraction (XRD) method was applied for detection of the lignocellulose crystallinity index (CrI) in the crude cell wall materials using Rigaku‐D/MAX instrument (Ultima III; Japan) as described by Zhang *et al*. (2013). The XRD method was detected with SD at ±0.05–0.15 using five representative samples in triplicate. The relative DP of cellulose was independently measured by the viscometry method as described by Zhang *et al*. (2013).

### Microscopic observations

Scanning electron microscopy (SEM; JSM‐6390/LV, Hitachi, Tokyo, Japan) was applied for observations of biomass residues and plant tissues obtained from pretreatments and sequential enzymatic hydrolysis as described by Li *et al*. (2015). For plant tissue *in situ* enzymatic digestion, the second‐stem transverse sections at heading stages were pretreated with 1% NaOH or 1% H_2_SO_4_ as described below, washed with distilled water until pH 7.0 and incubated with 1 g/L mixed cellulase for 2 h at 50 °C. After enzymatic hydrolysis, the tissue samples were sputter‐coated with gold and observed for 5–10 times with the photography of representative images. The mixed cellulase containing β‐glucanase (≥6 × 10^4^ U), cellulase (≥600 U) and xylanase (≥1.0 × 10^5^ U) was commercially available from Imperial Jade Bio‐technology Co., Ltd (Ningxia, 750002, China).

Transmission electron microscopy (TEM) was used to observe cell wall structures in the third leaf veins of three‐leave‐old seedlings. The samples were post‐fixed in 2% (w/v) OsO4 for 1 h after extensively washing in the PBS buffer and embedded with Super Kit (Sigma). Sample sections were cut with an Ultracut E ultramicrotome (Leica) and picked up on formvar‐coated copper grids. After poststaining with uranyl acetate and lead citrate, the specimens were viewed under a Hitachi H7500 transmission electron microscope.

AFM was applied to observe cellulose particles. The cellulose samples obtained as previously described in the ‘Cellulose extraction and gradation’ section were suspended in ultrahigh‐purity water and placed on mica using a pipette. The mica was glued onto a metal disc (15 mm diameter) after removal of extra water under nitrogen and then placed on the piezo scanner of AFM (MultiMode VIII; Bruker, Santa Barbara, CA). AFM imaging was carried out in ScanAsyst‐Air mode using Bruker ScanAsyst‐Air probes (tip radius, 2 nm; and silicon nitride cantilever; spring constant, 0.4 N/m) with a slow scan rate of 1 Hz. All AFM images were third‐flattened and analysed quantitatively using NanoScope Analysis software (Bruker). Ten dots of each AFM image were randomly selected, and the width (nm) of each dot was measured by NanoScope Analysis software (Bruker). The average particle width of each image was calculated from the selected ten particles.

### Biomass pretreatment and enzymatic hydrolysis

The chemical (H_2_SO_4_, NaOH) pretreatment and sequential enzymatic hydrolysis were performed as described by Xu *et al*. (2012). The CaO pretreatment was performed as follows: the well‐mixed biomass powder samples were treated with CaO (7.5% w/w) and shaken at 150 rpm for 36 h at 50 °C. SEM observation was described above using the biomass residues obtained from pretreatment and enzymatic digestion.

### Yeast fermentation and ethanol measurement

*Saccharomyces cerevisiae* (Angel yeast Co., Ltd, Yichang, China) was used in all the fermentation reactions, and the yeast powder was dissolved in 0.2 m phosphate buffer (pH 4.8) for 30 min for activation prior to use. The well‐mixed biomass powders were pretreated with CaO (7.5% w/w) and 1% H_2_SO_4_ as described above. After pretreatments, the biomass residues and supernatants were neutralized to pH 4.8 using appropriate amounts of CaO or H_2_SO_4_ and were autoclaved for 20 min. Then, mixed cellulases were loaded into each solution with the final enzyme concentration at 3.2 g/L (64 mg/g dry matter) and incubated at 50 °C under 150 rpm for 48 h. After that, the activated yeast was inoculated into the mixture of enzymatic hydrolysates and residues, and to the initial cell mass concentration at 0.5 g/L. The fermentation experiments were performed at 37 °C for 48 h, and the tube cover was loosened a bit to remove the generated CO_2_. The fermentation solution was distilled after 48 h for determination of ethanol content. All samples were carried out in the biological triplicates.

Ethanol content was measured using the dichromate oxidation method (Fletcher and van Staden, 2003) with minor modifications (Li *et al*., 2014).

### Calcofluor, CGA325′615 and MG132 treatments in the plant growth

The germinated seeds of *Osfc16* mutant and wild type were transferred onto the MS media supplied with Calcofluor White dye (Sigma‐Aldrich Co. LLC, California, USA) at different concentrations. After 24‐h incubation, the root tissues were measured every 24 h and harvested after 48 h for cellulose content, DP and CrI assays. For CGA325′615 (CGA) treatment, the germinated seeds were incubated with 20 nm CGA (kindly provided by Syngenta Com., Switzerland) in the MS media for 72 h. The root tissues were then measured and harvested for cellulose content, DP and CrI assays. All experiments were performed in the biological triplicates.

For the MG132 treatment, 6‐week‐old seedlings were incubated with 150 mm MG132 (dissolved in 1% DMSO; purchased from Alabiochem Tech. Co., Ltd, China) for 4 h. The seedlings were also treated with 1.5% DMSO as control. After treatments, total proteins of the seedlings were extracted in the extraction buffer (50 mm MOPS/NaOH buffer, pH 7.5, 0.25 m sucrose, 1.0 mm PMSF, 1.0 μm pepstatin A and 1.0 μm leupeptin), transferred to 15‐mL tubes and centrifuged at 2000 ***g*** for 10 min at 4 °C. The supernatant was incubated with 100 mm MG132 or the solvent DMSO for 1 h in room temperature. The protein concentration was determined using the BCA kits (Yeasen Tech. Co., Ltd, China). The reactions were stopped by the addition of SDS‐PAGE loading buffer.

### Microsomal membrane extractions

Microsomal membranes were extracted as described by Peng *et al*. (2002) using fresh rice stem tissues (14 g) at heading stage with minor modification. The samples were ground to a fine powder in liquid nitrogen and extracted with 70 mL of ice‐cold extraction buffer (50 mm MOPS/NaOH buffer, pH 7.5, 0.25 m sucrose) containing protease inhibitors (1.0 mm PMSF, 1.0 μm pepstatin A and 1.0 μm leupeptin). The extracts were transferred to 15‐mL tubes and centrifuged at 2000 ***g*** for 10 min at 4 °C. The resultant supernatant was filtered through two layers of gauze, and the filtrate was centrifuged at 100 000 ***g*** for 30 min. The remaining pellet was suspended in extraction buffer containing protease inhibitors and incubated for 30 min at 4 °C under continuous stirring in the presence of 0.05% digitonin. Finally, the homogenate was centrifuged at 5000 ***g*** for 15 min. The protein concentration in the supernatant was determined using the BCA kits (Yeasen Tech. Co., Ltd, China).

### Immunoprecipitation and Western blot analysis

Microsomal membrane extracts were suspended in the extraction buffer containing protease inhibitors and held under continuous stirring for 30 min at 4 °C in the presence of 2% Triton X‐100. The homogenates were then centrifuged at 5000 ***g*** for 15 min, and the extracted proteins were measured by the BCA kits as used at the same amounts in mutant and wild‐type plants. 500 μL supernatants (2% Triton X‐100 soluble) was mixed with 5 μL (9 μg) of anti‐CESA4/7 and incubated for 1 h at 4 °C. Next, 40 μL of protein A–agarose was added into sample tubes and gently shaken for 1 h at 4 °C with end‐over‐end rotation. After centrifugation for 1 min at 2000 ***g***, the harvested pellets were washed three times with ice‐cold extraction buffer and heated in 50 μL of sampling buffer at 70 °C for 5 min, then at 100 °C for 5 min. The obtained proteins were loaded into a 10% SDS‐PAGE gel.

Following electrophoresis separation, the proteins were transferred to a PVDF membrane. The membrane was blocked with TBS buffer (20 mm Tris‐HCl and 500 mm NaCl, pH 7.5) plus 5% nonfat dry milk for 1.5 h, rinsed with TTBS buffer (0.05% Tween‐20 in TBS) for three times and incubated with primary antibody serum (CESA4 antibody, 1 : 400 dilution; CESA7 antibody, 1 : 400 dilution; CESA9 antibody, 1 : 500 dilution) for 1 h at room temperature. Generations of CESA4‐, CESA7‐ and CESA9‐specific antibodies were described previously (Zhang *et al*., 2009). After three times of washing with TTBS, the membrane was incubated with secondary antibody (affinity‐purified phosphatase‐labelled goat anti‐rabbit IgG at a 1 : 10000) for 1 h at room temperature. The membrane was finally washed three times with TTBS and one time with TBS (200 mm Tris‐HCl, 150 mm NaCl, pH 7.5). The reactions were detected by the ECL Plus Western Blotting Detection. The relative protein levels were calculated using Quantity One software and the RuBisCO large subunit protein (RbcL) of SDS‐PAGE gel as internal reference.

## Supporting information


**Figure S1.** CESA9 mutation at the fully conserved CESA site in all CESA family proteins of plant species examined in rice, *Arabidopsis*, cotton, sorghum, maize, Brachypodium, poplar and Eucalyptus. ** indicates *Osfc16* mutation site.
**Figure S2.** Biomass enzymatic saccharification of mature stems in *Osfc16* and WT. Hexose yields released from time course enzymatic hydrolysis after (a) 1% NaOH or (b) 1% H_2_SO_4_ pretreatment.
**Table S1.** Information on CESA mutants and transgenic lines in plants.
**Table S2.** Agronomic traits in WT and *Osfc16* in the three paddy field experiments from 2012 to 2014.
**Table S3.** Agronomic traits of WT, *Osfc16* and complementary line in field experiment.
**Table S4.** Hexoses released from enzymatic (mixed‐cellulase) hydrolysis after pretreatments with NaOH and H_2_SO_4_.
**Table S5.** Ethanol yields obtained by yeast fermentation from biomass enzymatic hydrolysis of the mature stems after chemical pretreatments.
**Table S6.** Effects of Calcofluor and CGA on cellulose level, CrI and DP in WT and *Osfc16*.
**Table S7.** Cell wall composition (% dry matter) in WT and *Osfc16*.
**Table S8.** Cellulose DP of two gradated fractions in hull and stem of WT and *Osfc16*.
