# Supplementary material for: OsCESA9 conserved‐site mutation leads to largely enhanced plant lodging resistance and biomass enzymatic saccharification by reducing cellulose DP and crystallinity in rice
Source: Plant Biotechnol J. 2017 Mar 15;15(9):1093–104. doi: 10.1111/pbi.12700 (PMC5552474; doi:10.1111/pbi.12700)
Supplement: Supplementary file 1 — Figure S1. CESA9 mutation at the fully conserved CESA site in all CESA family proteins of plant species examined in rice, Arabidopsis, cotton, sorghum, maize, Brachypodium, poplar and Eucalyptus. ** indicates Osfc16 mutation site. Figure S2. Biomass enzymatic saccharification of mature stems in Osfc16 and WT. Hexose yields released from time course enzymatic hydrolysis after (a) 1% NaOH or (b) 1% H2SO4 pretreatment. Table S1. Information on CESA mutants and transgenic lines in plants. Table S2. Agronomic traits in WT and Osfc16 in the three paddy field experiments from 2012 to 2014. Table S3. Agronomic traits of WT, Osfc16 and complementary line in field experiment. Table S4. Hexoses released from enzymatic (mixed‐cellulase) hydrolysis after pretreatments with NaOH and H2SO4. Table S5. Ethanol yields obtained by yeast fermentation from biomass enzymatic hydrolysis of the mature stems after chemical pretreatments. Table S6. Effects of Calcofluor and CGA on cellulose level, CrI and DP in WT and Osfc16. Table S7. Cell wall composition (% dry matter) in WT and Osfc16. Table S8. Cellulose DP of two gradated fractions in hull and stem of WT and Osfc16. [file PBI-15-1093-s001.pdf]

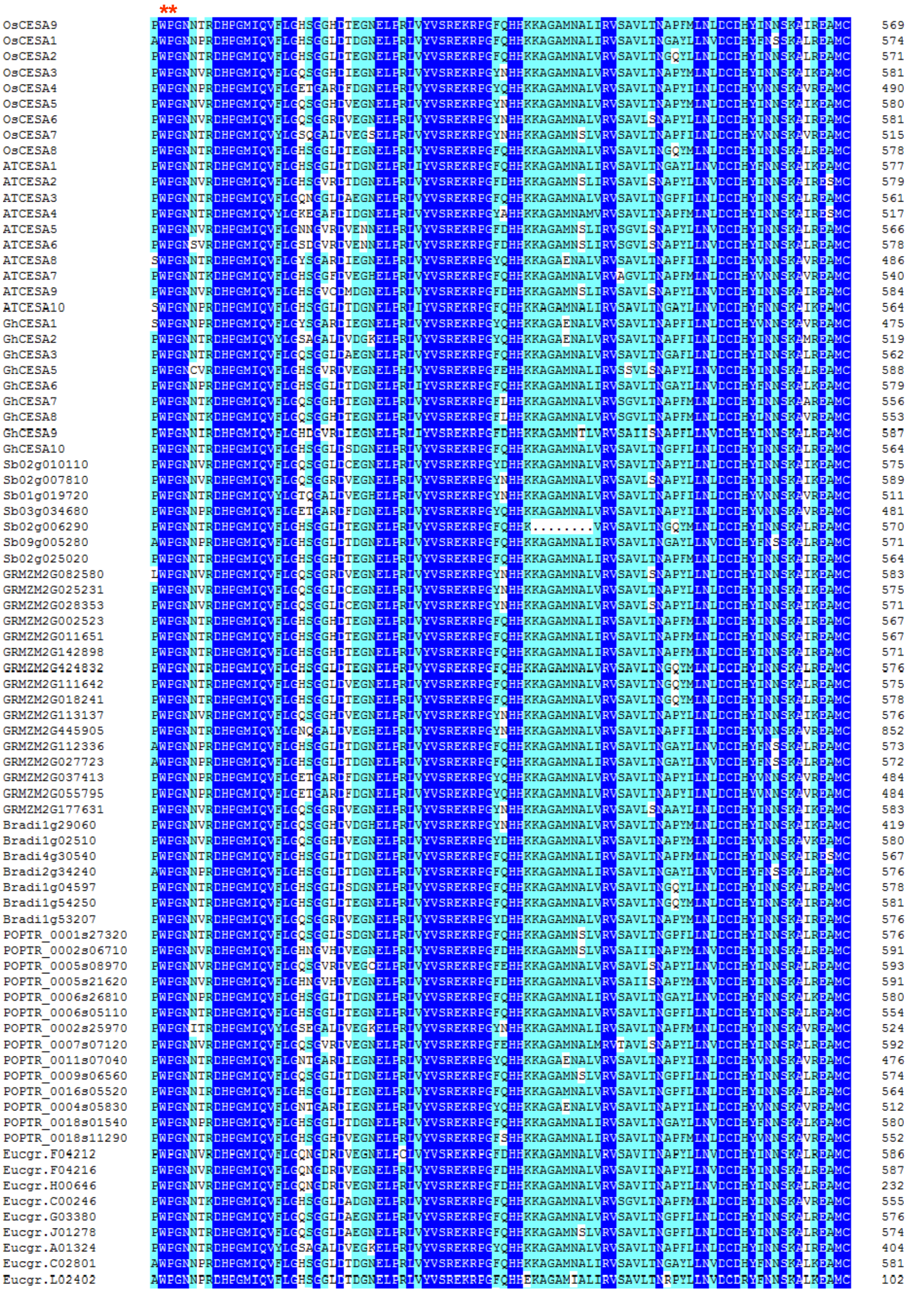

Figure S1. CESA9 mutation at the fully-conserved CESA site in all CESA family proteins of plant species examined in rice, *Arabidopsis*, cotton, sorghum, maize, brachypodium, poplar and Eucalyptus. ** indicate *Osfc16* mutation site.

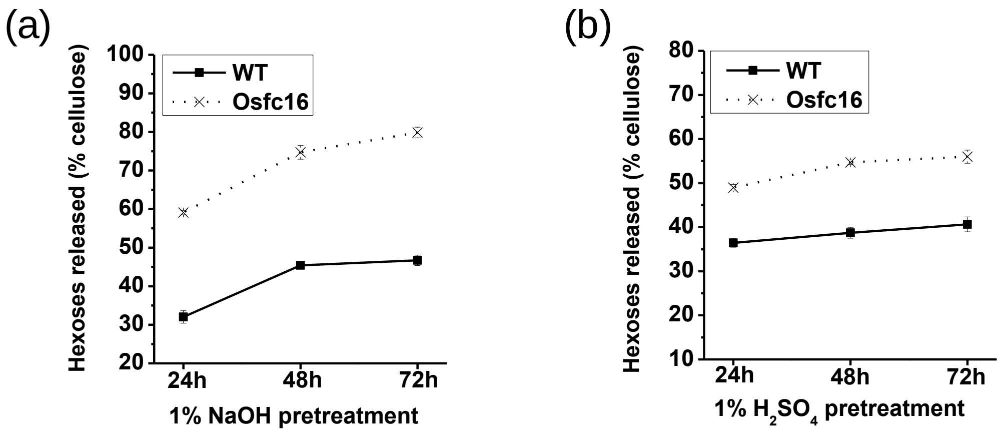

Figure S2. Biomass enzymatic saccharification of mature stems in *Osfc16* and WT. Hexoses yields released from time course enzymatic hydrolysis after (a) 1% NaOH or (b) 1% H_2SO_4 pretreatment.

Table S1. Information on CesA mutants and transgenic lines in plants

Forward genetics					
**Variety**	**Gene**	**Mutant Alleles**	**Phenotype**	**Ecotype**	**Reference**
**Arabidopsis**†	*AtCesA1*	*rsw1-1 (A549V)*	temperature sensitive, radial swelling, cellulose deficient, rosette disintegration	Col	(Arioli et al., 1998)
		rsw1-2 (G631S)	radial swelling, embryonic swelling, cellulose deficient	Ler	(Gillmor et al., 2002)
		rsw1-10 (splicing variant)	leaky allele, dwarf etiolated hypocotyls, cellulose deficiency	Ws	(Fagard et al., 2000a)
		rsw1-20 (D780N)	short, swollen etiolated hypocotyls and cotyledons, cell division defects, cell wall defects	Ler	(Beeckman et al., 2002)
		rsw1-45 (E779K)	short, swollen etiolated hypocotyls and cotyledons, cell division defects, cell wall defects	Ler	(Beeckman et al., 2002)
		aegeus (A903V)	quinoxyphen resistant, reduced cellulose content, altered cellulose crystallinity, increased CSC velocity	Col	(Harris et al., 2012)
		any1 (D604N)	short, swollen roots, dwarf plants, fragile trichome, no cellulose content difference, but reduced cellulose crystallinity, reduced CSC velocity	Col	(Fujita et al., 2013)
	AtCesA3	*ixr1-1 (G998D)*	resistance to isoxaben	Col	(Scheible et al., 2001)
		ixr1-2 (T942I)	resistance to isoxaben	Col	(Scheible et al., 2001)
		cev1 (G617E)	short roots, dwarf plant, increased jasmonate and ethylene production, constitutive stress response, cellulose content deficiency	Col	(Ellis et al., 2002)
		eli1-1 (S301F)	short, swollen root and etiolated hypocotyl, dwarf plant, ectopic lignification, reduced cellulose synthesis, activated defense	Col	(Brown, 1999b; CanoDelgado et al., 2003)
		eli1-2 (A522V)	responses (jasmonate and ethylene)	Col	(Brown, 1999b; CanoDelgado et al., 2003)
		rsw5 (P1056S)	temperature sensitive, radial swelling, dwarf plants, cellulose deficiency	Col	(Wang et al., 2006)
		thanatos (P578S)	semi-dominant allele, short, swollen root and hypocotyl, cellulose deficiency	Col	(Daras et al., 2009)
		mre (G916E)	short, swollen root and etiolated hypocotyl, cellulose deficiency, dwarf plants	Col	(Pysh et al., 2012)
	AtCesA4	*irx5-1*	thin, irregular xylem and interfascicular cell walls, cellulose content deficiency, dwarf plants	Ler	(Taylor et al., 2003)
		irx5-2 (W995stop)		Ler	(Taylor et al., 2003)
		irx5-3 (Q263stop)		Ler	(Taylor et al., 2003)
		exi2 (Y939stop)	small rosette leaves, flowers, siliques, reduced cell elongation, dwarf plants, altered vasculature and cell morphology, cellulose content deficontent deficiency, impaired water transport	Ler	(Rubio-Diaz et al., 2012)
	AtCesA6	*prc1-1 to prc1-12*	short etiolated hypocotyls and roots, incomplete cell walls, cellulose deficiency	Col/Ws	(Fagard et al., 2000a)
		ixr2-1 (R1064W)	semi-dominant allele, resistance to isoxaben	Col	(Desprez et al., 2002)
		prc1-20 (52-isx)	oryzalin hypersensitive, isoxaben hypersensitive, short, swollen etiolated hypocotyls and roots, cellulose content deficiency	Col	(Paredez et al., 2008)
	AtCesA7	*irx3 (W859stop)*	irregular, collapsed xylem, cellulose deficiency	Ler	(Taylor et al., 1999)
		fra5 (P557T)	semi-dominant allele, reduced fiber cell wall thickness, cellulose content deficiency, dwarf plants, short roots and etiolated hypocotyls	Col	(Zhong et al., 2003)
		mur10-1 (W444stop)	collapsed xylem, dwarf aerial organs, reduced female fertility, reduced tensile strength in hypocotyls, altered primary cell wall	Col	(Bosca et al., 2006)
		mur10-2 (H734Y)	composition	Col	(Bosca et al., 2006)
		exi5 (W954stop)	small rosette leaves, flowers, siliques, reduced cell elongation, dwarf plants, altered vasculature and cell morphology, cellulose content deficiency, impaired water transport	Ler	(Rubio-Diaz et al., 2012)
	AtCesA8	*irx1-1 (D683N)*	irregular, collapsed xylem, cellulose deficiency, dwarf plants	Ler	(Taylor et al., 2000)
		irx1-2 (S679L)	irregular, collapsed xylem, cellulose deficiency, dwarf plants	Ler	(Taylor et al., 2000)
		lew2-1 (W217stop)	increased tolerance to drought and osmotic stress, increased proline, soluble sugars, and abscisic acid content, collapsed	C24	(Chen et al., 2005)
		lew2-2 (L792F)	xylem, content deficiency cellulose content deficiency	Col	(Chen et al., 2005)
		fra6 (R362K)	reduced fiber cell wall thickness, reduced cellulose content	Col	(Zhong et al., 2003)
		exi1-1 (splicing variant)	small rosette leaves, flowers, siliques, reduced cell elongation, dwarf plants, altered vasculature and cell morphology, cellulose	Ler	(Rubio-Diaz et al., 2012)
		exi1-2 (G508E)	content deficiency, impaired water transport	Ler	(Rubio-Diaz et al., 2012)

Forward genetics					
**Variety**	**Gene**	**Mutant Alleles**	**Phenotype**	**Ecotype**	**Reference**
**Rice**	*OsCesA4*	*NE1031 (Tos17 insertion in the 6th exon)*	dwarf plants, brittle culm, cellulose deficiency, reduced fiber cell wall		(Tanaka et al., 2003)
		ND5658 (Tos17 insertion in the 6th exon)	thickness		(Tanaka et al., 2003)
		bc11 (G858R)	dwarf plants, brittle culm, cellulose deficiency, reduced wall thickness and structure, DCB resistance	NPB	(Zhang et al., 2009)
	OsCesA7	*NC0259 (Tos17 insertion in the 5th exon)*	dwarf plants, brittle culm, cellulose deficiency, reduced fiber cell wall		(Tanaka et al., 2003)
		ND8759 (Tos17 insertion in the 7th exon)	thickness		(Tanaka et al., 2003)
	OsCesA9	*ND2395 (Tos17 insertion in the 6th exon)*	dwarf plants, brittle culm, cellulose deficiency, reduced fiber cell wall		(Tanaka et al., 2003)
		NF1011 (Tos17 insertion in the 9th exon)	thickness		(Tanaka et al., 2003)
		BC6 (R588G)	semi-dominant allele, brittle culm, normal phenotype similar to WT, reduced cellulose content, incresased hemicellulose	IR68	(Kotake et al., 2011)
		S1-60 (G905D)	dwarf plants, brittle culm, cellulose deficiency, reduced wall thickness	NPB	(Wang et al., 2012)
		bc13 (G101K)	normal phenotype similar to wt, brittle culm, Cd tolerance, reduced cellulose content, slightly reduced secondary wall thickness	Yinhuazhan	(Song et al., 2014)
**Barley**	*HvCesA4*	*fs2 (964-bp insertion in the first intron)*	brittle culm, cellulose deficiency	Ohichi/Shiroseto	(Burton et al., 2010)

Reverse genetics					
**Genes**	**Construct**	**Variety**	**Host**	**Phenotype**	**Reference**
AtCesA3 ixr1-2	proM24-GFP::AtCESA3^{ixr1-2}	Tobacco	Wild type	dwarf plants, isoxaben resistance,aberrant spatial distribution of lignified secondary cell wall tissue, cellulose reduce, higher biomass digestion	(Sahoo et al., 2013)
AtCesA1	proAtCesA1::AtCesA1 (phosphorylation sites mutation)	Arabidopsis	*rsw1*	alter anisotropic cell expansion and cellulose synthesis in rapidly expanding roots and hypocotyls, rescued the temperature sensitive *rsw1* phenotype	(Chen et al., 2010)
AtCesA7	pro35S::AtCesA7	Arabidopsis	Wild type	no obvious phenotype	(Zhong et al., 2003)
AtCesA7 fra5	pro35S::AtCesA7^{fra5}	Arabidopsis	Wild type	dwarf plants, reduced primary and secondary wall thickness, reduced cellulose content and cell elongation	(Zhong et al., 2003)
AtCesA8 fra6	pro35S::AtCesA8^{fra6}	Arabidopsis	Wild type	no observable changes in the elongation of cells and organs	(Zhong et al., 2003)
AtCesA3	pro35S::AtCesA3	Arabidopsis	*rsw1*	could not complement the *rsw1* phenotype	(Burn et al., 2002)
AtCesA2	proAtCesA6::AtCesA2	Arabidopsis	*prc1-1*	only partially complemented *cesa6* mutant phenotype	(Desprez et al., 2007; Persson et al., 2007)
AtCesA5	proAtCesA6::AtCesA5	Arabidopsis	*prc1-1*		(Desprez et al., 2007; Persson et al., 2007)
HvCesA1	pro35S::HvCesA1	Barley	Wild type (Golden promise)	no visual abnormalities	(Tan et al., 2015)
HvCesA2	pro35S::HvCesA2	Barley	Golden promise	no visual abnormalities	(Tan et al., 2015)
HvCesA6	pro35S::HvCesA6	Barley	Golden promise	no visual abnormalities	(Tan et al., 2015)
HvCesA4	pro35S::HvCesA4	Barley	Golden promise	Dwarfism and died at an early stage	(Tan et al., 2015)
HvCesA8	pro35S::HvCesA8	Barley	Golden promise	Dwarfism and died at an early stage	(Tan et al., 2015)
BdCesA4	proUBI::amiR-CESA4	Brachypodium distachyon	Wild type	dwarf plants, delayed in inflorescence emergence, cellulose deficiency, reduced cell wall thickness	(Handakumbura et al., 2013)
BdCesA7	proUBI::amiR-CESA7	Brachypodium distachyon	Wild type	dwarf plants, delayed in inflorescence emergence, cellulose deficiency, reduced cell wall thickness	(Handakumbura et al., 2013)
PtdCesA8	pro35S::PtdCesA8	Aspen	Wild type	co-suppression, dwarf plants, massive reduction in cellulose, cell wall irregularities	(Joshi et al., 2011)

† Modify from Li et al. (2014)

References for Table S1.

1. T. Arioli et al., *Science* 279, 717-720 (1998).
2. C. S. Gillmor et al., *J. Cell Biol.* 156, 1003-1013 (2002).
3. M. Fagard, H. Hofte, S. Vernhettes, *Plant Physiol. Biochem.* 38, 15-25 (2000).
4. T. Beeckman et al., *Plant Physiol.* 130, 1883-1893 (2002).
5. D. M. Harris et al., *Proc. Natl. Acad. Sci. U.S.A.* 109, 4098-4103 (2012).
6. M. Fujita et al., *Plant Physiol.* 162, 74-85 (2013).
7. W. R. Scheible et al., *Proc. Natl. Acad. Sci. U.S.A.* 98, 10079-10084 (2001).
8. C. Ellis et al., *Plant Cell* 14, 1557-1566 (2002).
9. R. M. Brown, *Abstracts of Papers of the American Chemical Society* 218, U214-U214 (1999).
10. A. Cano-Delgado et al., *Plant J.* 34, 351-362 (2003).
11. J. wang et al., *Plant Physiol.* 142, 685-695 (2006).
12. G. Daras et al., *New Phytol.* 184, 114-126 (2009).
13. L. Pysh et al., *Physiol. Planta.* 144, 369-381 (2012).
14. N. G. Taylor et al., *Proc. Natl. Acad. Sci. U.S.A.* 100, 1450-1455(2003).
15. S. Rubio-Diaz et al., *PloS one* 7 (2012).
16. T. Desprez et al., *Plant Physiol.* 128, 482-490 (2002).
17. A. R. Paredez et al., *Plant Physiol.* 147, 1723-1734 (2008).
18. N. G. Taylor., *Plant Cell* 11, 769-780 (1999).
19. R. Zhong., *Plant Physiol.* 132, 786-795 (2003).
20. S. Bosca et al., *Plant Physiol.* 142, 1353-1363 (2006).
21. N. G. Taylor, S. Laurie, S. R. Turner, *Plant Cell* 12, 2529-2540 (2000).
22. Z. Chen et al., *Plant J.* 43, 273-283 (2005).
23. K. Tanaka et al., *Plant Physiol.* 133, 73-83 (2003).
24. B. Zhang et al., *Plant Mol. Biol.* 71, 509-524 (2009).
25. T. Kotake et al., *J. Exp. Bot.* 62, 2053-2062 (2011).
26. D. Wang et al., *Plant Sci* 196, 117-124 (2012).
27. X. Q. Song et al., *Mol. Plant* 6, 768-780 (2013).
28. R. A. Burton et al., *Plant Physiol*. 153, 1716-1728 (2010).
29. D. K. Sahoo, J. Stork, S. DeBolt, I. B. Maiti, *Plant Biotechnol. J.* 11, 362-372 (2013).
30. S. Chen, D. W. Ehrhardt, C. R. Somerville, *Proc. Natl. Acad. Sci. U.S.A.* 107, 17188-17193 (2010).
31. R. Zhong et al., *Plant Physiol.* 132, 786-795 (2003).
32. J. E. Burn, C. H. Hocart, R. J. Birch, A. C. Cork, R. E. Williamson, *Plant Physiol*. 129, 797-807 (2002).
33. T. Desprez et al., *Proc. Natl. Acad. Sci. U.S.A*. 104, 15572-15577 (2007).
34. S. Persson et al., *Proc. Natl. Acad. Sci. U.S.A*. 104, 15566-1557 (2007).
35. Tan et al., *BMC Plant Biology* 15, 62 DOI 10.1186/s12870-015-0448-y (2015)
36. P. P. Handakumbura et al., *BMC Plant Biol*. 13, 131 (2013).
37. C. P. Joshi et al., *Mol. Plant* 4, 331-345 (2011).
38. S. Li, L. Bashline, L. Lei, Y. Gu, The Arabidopsis book / American Society of Plant Biologists 12, e0169 (2014).

Table S2. Agronomic traits in WT and *Osfc16* in the three paddy field experiments from 2012 to 2014

	2012			2013			2014		
	**WT**	***Osfc16***		**WT**	***Osfc16***		**WT**	***Osfc16***	
**Lodging index**	71.32±13.96	54.33±9.79*	-24%†	74.76±10.42	57.7±5.31**	-23%	84.28±4.36	66.63±6.35**	-18%
**Dry straw (g/plant)**	34.89±2.98	43.59±6.66*	25%	37.09±3.01	52.25±4.12**	41%	37.77±2.62	48.78±1.88**	29%
**Tillers/plant**	22±3.87	35±1.97**	59%	22±2.67	37±2.31**	68%	20±1.27	32±2.52**	60%
**Plant height (cm)**	102.80±4.80	89.40±8.95**	-13%	102±6.37	90±7.86*	-12%	95.96±4.58	80.98±1.12**	-16%
**1,000-Grain weight (g)**	24.57±0.25	24.16±0.08	0%	24.84±0.15	24.66±0.19	0%	24.53±0.51	24.24±0.19	0%
**Dry spike (g/plant)**	39.50±5.63	39.27±3.4	0%	41.93±3.69	41.30±3.49	0%	38.67±2.04	38.04±2.29	0%

† indicated the increased or decreased (-) percentages by subtraction of the values between mutant and WT divided by WT.

* and ** indicated the significant difference between *Osfc16* mutant and WT by *t*–test at $p < 0.05$ and 0.01, respectively.

Table S3. Agronomic traits of WT, *Osfc16* and complementary line in field experiment

	WT	*Osfc16*	Complementary line
**Lodging index**	84.28±4.36	66.63±6.35**	78.94±7.74
**Dry straw (g/plant)**	37.77±2.62	48.78±1.88**	39.69±1.74
**1,000-Grain weight (g)**	24.53±0.51	24.24±0.19	24.31±0.36

** indicated significant difference between *Osfc16* mutant and WT/complementary line by *t*–test at $p < 0.01$ (n=3) from three independent biological duplications.

Table S4. Hexoses released from enzymatic (mixed-cellulase) hydrolysis after pretreatments with NaOH and H_2SO_4

	Hexoses released (% cellulose)											
	0.5% NaOH		1% NaOH		4% NaOH		0.5% H_2SO4		1% H_2SO4		2% H_2SO4	
WT	23.5±0.4	109%†	45.4±0.7	64%	74.2±0.9	30%	33.1±1.1	54%	38.7±1.2	41%	41.4±1.4	41%
Osfc16	49.2±1.3**		74.7±1.8**		96.4±1.1**		51±0.6**		54.7±0.5**		58.6±0.6**	

† indicated the increased percentages by subtraction of the values between mutant and WT divided by WT.

** indicated the significant difference between *Osfc16* mutant and WT by *t*–test at $p < 0.01$.

Table S5. Ethanol yields obtained by yeast fermentation from biomass enzymatic hydrolysis of the mature stems after chemical pretreatments

	Ethanol yield (% total hexoses)				Ethanol yield (g/plant)			
	7.5% CaO		1% H_2SO4		7.5% CaO		1% H_2SO4	
WT	24.1±0.7		29.7±0.7		4.3±0.1		5.3±0.1	
		34%[†]		33%		42%		42%
Osfc16	32.5±0.3**		39.6±1.2**		6.1±0.1**		7.5±0.2**	

[†] indicated the increased percentages by subtraction of the values between mutant and WT divided by WT.
** indicated the significant difference between *Osfc16* mutant and WT by *t*–test at $p < 0.01$.

Table S6. Effects of Calcofluor and CGA on cellulose level and CrI in WT and *Osfc16*

		Cellulose (% dry matter)		Cellulose CrI[†] (%)	
WT	Control	29.6±0.1	-32%[‡]	33.6	-23%
	Calcofluor	20.2±0.3**		26	
Osfc16	Control	26±0.1	-17%	32	-10%
	Calcofluor	21.7±0.3**		28.8	
WT	Control (DMSO)	27.8±0.2	-31%	30	-33%
	CGA	19.1±0.5**		20.2	
Osfc16	Control (DMSO)	24.4±0.4	-17%	27	-7%
	CGA	20.3±0.1**		25.1	

[†] CrI was measured using crude cell walls of biomass samples, and CrI method was examined with SD values ranged from 0.05 to 0.15 using five representative samples in technique triplicates.

[‡] Percentage of the increased or decreased (-) level calculated by subtraction of the values between the Calcofluor/CGA and control divided by control.

****** indicated significant difference between the pairs by *t*–test at $p < 0.01$; Data of cellulose levels from three independent biological duplications。

Table S7 . Cell wall composition (% dry matter) in WT and *Osfc16*

		Cellulose[&]	Hemicelluloses	Lignin	Uronic acids
**Hull***	**WT**	33.47 ± 1.35	23.85 ± 0.41	20.72 ± 0.29	0.16 ± 0.01
	Osfc16	20.63 ± 0.25	29.91 ± 0.59	25.72 ± 0.31	0.22 ± 0.02
**Stem**	**WT**	23.43 ± 0.38	17.47 ± 0.34	7.99 ± 0.15	0.51 ± 0.03
	Osfc16	20.80 ± 0.47	20.67 ± 0.30	9.67 ± 0.19	0.54 ± 0.02

* Hull and stem samples were obtained from mature tissues as described in Figure 6a;

& Wall polymers level as mean ± SD (n = 3) from three independent biological duplications.

Table S8. Cellulose DP of two gradated fractions in hull and stem of WT and *Osfc16*

Cellulose fraction		Hull	Stem
**High-DP[†] cellulose**	**WT**	1027-1079[@] (34%-40%)[*]	1077-1102 (10%-19%)
	Osfc16	778-782 (33%-34%)	644-752 (18%-26%)
**Low-DP cellulose**	**WT**	680-767 (60%-66%)	407-504 (81%-90%)
	Osfc16	612-684 (66%-67%)	423-540 (74%-82%)

[@] DP values were derived from two independent biological experiments determined by viscometry method.

[†] Purified cellulose was gradated into two fractions as high- and low-DP cellulose described in "Methods" section.

***** High- or low-DP cellulose percentage of total cellulose level determined by anthrone/H_2SO_4 method.
